# Dataset for Genome Sequencing and De Novo Assembly of the Candidate Phyla Radiation in Supragingival Plaque

**DOI:** 10.1155/2022/4899824

**Published:** 2022-03-19

**Authors:** Song Jiang, Jie Nie, Yuxing Chen, Shiying Zhang, Xiaoyan Wang, Feng Chen

**Affiliations:** ^1^Department of Endodontics, Peking University School and Hospital of Stomatology, Beijing 100081, China; ^2^Central Laboratory, Peking University School and Hospital of Stomatology, Beijing 100081, China

## Abstract

The Candidate Phyla Radiation (CPR), as a newly discovered and difficult-to-culture bacterium, accounts for the majority of the bacterial domain, which may be related to various oral diseases, including dental caries. Restricted by laboratory culture conditions, there is limited knowledge about oral CPR. Advances in metagenomics provide a new way to study CPR through molecular biology. Here, we used metagenomic assembly and binning to reconstruct more and higher quality metagenome-assembled genomes (MAGs) of CPR from oral dental plaque. These MAGs represent novel CPR species, which differed from all known CPR organisms. Relative abundance of different CPR MAGs in the caries and caries-free group was estimated by mapping metagenomic reads to newly constructed MAGs. The relative abundance of two CPR MAGs was significantly increased in the caries group, indicating that there might be a relationship with caries activity. The detection of a large number of unclassified CPR MAGs in the dataset implies that the phylogenetic diversity of CPR is enormous. The results provide a reference value for exploring the ecological distribution and function of uncultured or difficult-to-culture microorganisms.

## 1. Introduction

The human oral cavity, as one of five major microecological systems in the human body, has been used as the main model system for microbial community research to understand microbial ecology and function [1]. The data from the Human Microbiome Project [2] show that the oral cavity can contain up to 700 types of microorganisms, 30% of which are uncultured. Oral microorganisms include bacteria, fungi, viruses, and bacteriophages. Oral bacteria, which are relatively abundant and easy to be detected and cultured, have always been a hot topic in the study of oral microorganisms. In recent years, although we have made great progress in understanding the complex microbial communities in the oral cavity, the research on some microorganisms is still not deep enough, for example, a new bacterial group called “Candidate Phyla Radiation” (CPR) has not been fully studied. With regard to such microorganisms, studies have shown that they may be associated with a variety of oral diseases. For example, the Saccharibacteria bacterium (once known as TM7), belonging to CPR organisms, is widespread in the human oral microbiota, and there is increasing evidence that they are associated with a variety of mucosal diseases, including periodontitis, halitosis, and inflammatory bowel disease [3–6]. TM7 may have a certain effect on the microecology of oral flora, playing a vital but little-known role in the occurrence and development of oral diseases.

Caries is the most common and frequently occurring chronic oral infectious disease, which is characterized by progressive destruction of hard tissues of teeth. The aetiological mechanism of caries has always been a hot research topic and the basis for caries treatment and prevention. The four-factor theory of caries considers that cariogenic bacteria, susceptible host, appropriate substrate, and time are necessary conditions for caries [7], and cariogenic bacteria is the most important link. Now, most scholars tend to agree with the “ecological plaque hypothesis” [8], believing that the bacteria with the so-called “cariogenic” are by no means limited to one or some bacteria, and the bacterial community is considered as a whole. These bacteria are originally the resident flora existing in the oral cavity, and numerous microorganisms maintain close contact with each other, thus maintaining the demineralization and remineralization on the tooth surface as a whole through complex interaction. When the microenvironment changes, one or more microflora in dental plaque obtains a competitive advantage and breaks the ecological balance between microorganisms, between dental plaque and tooth surface, and between the oral environment, thus leading to the occurrence of dental caries [9]. As a kind of difficult-to-cultivate and poorly understood bacteria, whether the CPR organisms acting as the aetiological factor of caries, or the type of the CPR organisms changed from health to disease states is still unknown.

Unlike fungi and viruses, CPR is a recently discovered species of bacterial organisms that has greatly influenced our perception of the diversity of life on Earth [10]. This previously unknown bacterium may contain a total of more than 70 phyla, accounting for more than 25% of the bacterial domain [11]. CPR organism is found in a variety of niches and has similar characteristics, such as containing self-splicing introns of 16S subunit ribosomal RNA (16S rRNA) genes and archaeal-specific RuBisCO genes [12], lacking genes that can be used to encode the CRISPR/Cas phage defence system [13], etc. Due to the reduction of the genome, their biosynthetic and metabolic capacities are limited, and they have no electron transfer chains, tricarboxylic acid cycles, amino acid and membrane biosynthetic pathway, and various ribosome subunits [14–19]. High-resolution frozen transmission electron microscopy shows that the CPR cell size is 0.009 ± 0.002 mm^3^, which is consistent with the size of their small genome [18]. To sum up, these shared properties indicate that members of the CPR may show a symbiotic lifestyle and rely on essential metabolites of partner cells, while potentially providing unstable fermentation (e.g., acetate) in return [15, 20–22]. This codependent lifestyle may explain their resistance to culture in vitro [23]. However, it is unclear whether environmental conditions change from health status to caries will affect the composition of CPR organisms.

The challenges of cultivating CPR limit research on their phylogeny and functional diversity. With the development of bioinformatics analyses and high-throughput sequencing, more and more metagenome sequencing data have been obtained from diverse ecological niches and multiple parts of the human body including the oral cavity. Metagenomics is a DNA sequencing methodology based on shotgun sequencing, which sequences the DNA directly separated from the environment and then, assigns the reconstructed genome segments to the genome sketches [24]. This polymerase chain reaction (PCR)-independent genomic (rather than gene)-based approach is valuable for researchers to overcome the obstacles described above and provide information about the metabolic potential of uncultured organisms. In addition, there are various phylogenetically informative sequences that can be used to classify CPR organisms.

Here, we used metagenome shotgun sequencing data obtained from the study [25] for metagenomic analysis. Limited to the memory of the server, only 25% raw data were assembled and analysed downstream in the original study. However, we used all the raw data to characterize the distribution, abundance, and functional differences of CPR organisms in supragingival biofilm swabs of twin pairs, including caries and healthy children using shotgun metagenomic sequencing, de novo assembly, and binning techniques. Previous metagenomic data analysis was incomplete, and data mining was insufficient. Our work utilizes resources of public databases to conduct in-depth metagenomic analysis to obtain more valuable microbial information is significant for metagenomic research.

## 2. Materials and Methods

### 2.1. Metagenomic Datasets

The individual reads of 88 subjects were downloaded from the NCBI SRA database, with accession numbers SRR6865436 to SRR6865523. In the original study [25], International Caries Detection and Assessment System (ICDAS II) criteria were used for caries assessment. Subjects with either enamel caries or dentinal caries are referred to as diseased, while subjects without enamel or dentinal caries are referred to as healthy, unless otherwise stated. Dental plaque samples were collected, and DNA was extracted according to a previously published method [26]. The metagenomic libraries were constructed using the NEBNext Illumina DNA library preparation kit (New England Biolabs, Ipswich, MA) and then were sequenced for 300 cycles using the Illumina NextSeq 500 High-Output kit according to standard manufacturer's specifications (Illumina Inc., La Jolla, CA).

### 2.2. Data Quality Control

Sequence data quality was assessed using FastQC v0.11.7 (https://www.bioinformatics.babraham.ac.uk/projects/fastqc/). The raw reads from each sample were subjected to adapter trimming and low-quality filtering using Trimmomatic v0.36 [27] with parameters of “LEADING : 3 TRAILING : 3 SLIDINGWINDOW : 4 : 15 MINLEN : 36.” Each sample's reads were then aligned to human genome build hg38 using Bowtie2 (v2.3.5.1) [28] to filter any genes that were of host origin and avoid host contamination. FastQC was performed again to evaluate the quality of the remaining reads.

### 2.3. De Novo Assembly and Binning

Total cleaned reads were first assembled on a per sample basis using the metaSPAdes v3.14.0 [29] with option--meta and default parameters [30]. The required coverage depth for the binning was inferred by mapping the raw cleaned reads back to their assemblies using Bowtie2 (v2.3.5.1) and then, calculating the corresponding read depths of each individual contig using SAMtools v1.9 [31] (“samtools view-Sbu” followed by “samtools sort”) together with the jgi_summarize_bam_contig_depths function from MetaBAT 2 (v2.12.1) [32]. The MetaBAT 2 program was then called using a minimum contig length threshold of 1,500 bp (option--minContig 1500) and default parameters (minCV 1.0, minCVSum 1.0, maxP 95%, minS 60, and maxEdges 200), leading to the generation of 424 bins covering 3,408,272,494 bases.

### 2.4. Taxonomic Analyses and Phylogenetic Analysis

We used Kraken2 v2.0.8 [33] to classify the contigs with default parameters according to the Kraken2 database, and each genome bin was assigned the lowest taxonomic label that was assigned to at least 70% of the contigs in the genome bin. Twenty-nine CPR genome bins were recovered and the completeness and contamination of each bin were estimated with CheckM v1.1.2 [34] using the lineage_wf workflow. Thirteen low-quality (<50% completeness) genome bins were removed, and the remaining sixteen CPR genome bins were used for downstream analysis as metagenome-assembled genomes (MAGs). 16S SSU rRNA genes reads were extracted from bins using the command–ssu_finder of CheckM v1.1.2 with default parameters. 16S SSU rRNA genes reads of 22 representative CPR genomes (including sixteen TM7; three Gracilibacteria; and three Absconditabacteria) were downloaded from the expanded Human Oral Microbiome Database (eHOMD) (https://www.homd.org). The alignment of total 16S SSU rRNA was conducted using the ClustalW method. The misaligned ends and regions with >95% gaps were trimmed, and the final alignment was used to generate a 16S rRNA phylogenetic tree by using the maximum-likelihood method through the MEGA7 software [35], with a bootstrap test of 1000 replicates. Firstly, a JTT model was used to estimate a matrix of pairwise distances, then to which applied Neighbor-Join and BioNJ algorithms, obtaining the initial tree for the heuristic search automatically. Secondly, a tree with the highest log-likelihood was constructed by choosing the topology with the highest log-likelihood value. At last, the branch lengths were adjusted with the number of substitutions per site.

### 2.5. Comparative Analysis of MAGs

Five MAGs for CPR were downloaded from the original literature, including three TM7 MAGs and two GN02 MAGs. Quast v5.0.2 [36] was used to determine the quality of the sixteen newly assembled genomes and five original literature's genomes for further comparison. Then, pairwise average nucleotide identity (ANI) values of newly assembled genomes and the original literature's genomes were calculated by using JSpecies (https://imedea.uib-csic.es/jspecies/) [37]. Whole-genome comparisons were conducted by aligning sequenced genomes with Progressive MAUVE v2.4.0 [38], using the MAUVE Multiple Genome Alignment software (https://asap.ahabs.wisc.edu/mauve/).

As reference genome, each individual assembled MAG was indexed using Bowtie2 v2.3.5.1 with the command “bowtie2‐build.” Clean reads of the caries group and the caries-free group were aligned to the reference genomes, respectively, with Bowtie2 v2.3.5.1. The resulting count tables were converted to include length, coverage, and relative abundance in the measurements by adjusting the transcript per million (TPM) [39] calculation for the contigs, a fundamental standardization of metagenomic assembly, as the contigs length has an inherently wide distribution. The relative abundance for each CPR MAG in the caries and caries-free group was calculated, and the differences in relative abundance were analysed using Fisher's exact test with Benjamini–Hochberg False Discovery Rate (FDR) correction using STAMP v2.1.3 [40]. A *p* value <0.01 was taken to denote statistical significance.

### 2.6. Metagenomic Annotation and Functional Characterization

The significantly increased MAGs in the caries group were selected. The genes from these MAGs were predicted using Prodigal v2.6.3 [41]. Predicted protein sequences were annotated against KEGG with GhostKOALA (genus_prokaryotes + family_eukaryotes) (https://www.kegg.jp/ghostkoala/) [42]. Marker genes for central metabolic pathways and key environmental element transformations were identified based on K number assignments. The metabolic capacity of MAG is determined by the similarity of gene content with the KEGG genome with known functions and the presence of key genes and pathways in MAG. KEGG ids extracted from the MGENE group associated with each KEGG genome (a total of 5647 comparisons) were added to the matrix of all MAG KEGG ids, respectively. The functional ability of MAG was evaluated by using the functional ability of up to four nearest KEGG genomes.

## 3. Results and Discussion

### 3.1. Data Overview

In the original literature, the shotgun sequencing of metagenome was conducted on 88 dental plaque samples from 44 twin pairs including 50 from caries patients, and 38 from healthy persons (the information for the samples is shown in Table S1). We downloaded the SRA files with serial accession numbers from SRR6865436 to SRR6865523 (BioProject accession number PRJNA383868) and converted them to FASTQ files using SRA Toolkit 2.9.6 (https://www.ncbi.nlm.nih.gov/sra). A total of 96 Gb of paired-end sequence data were generated, and each sample had an average of 5.52 million reads (1.1 Gb). After filtering low-quality and human reads, about 47.8% of the sequence reads remained. The remaining, quality-filtered reads were assembled with metaSPAdes produced contigs that could undergo genomic binning by MetaBAT 2, generating a total of 424 bins covering 3,408,272,494 bases.

### 3.2. More Discoveries of Uncultured CPR Organisms

Having identified 424 bins in the dental plaque, we sought to determine their taxonomic classification and evaluated their quality. By complementing the phylogenetic inference method of CheckM with exact alignment based on k-mers using Kraken2 against the Kraken2 database, we attempted to assign the most likely taxonomic lineage to each bin. We screened 29 CPR bins, but 13 of them were of poor quality (<50% completeness) and were removed. The remaining 16 CPR genome bins were used for downstream analysis as MAGs including thirteen genomes for the TM7 lineage and three genomes for the Gracilibacteria (GN02) lineage (see Table 1). Of these MAGs, eight were high-quality genomes (>90% completeness), and eight were medium-quality genomes (<90% completeness and >50% completeness) as estimated by CheckM.

**Figure 1:**
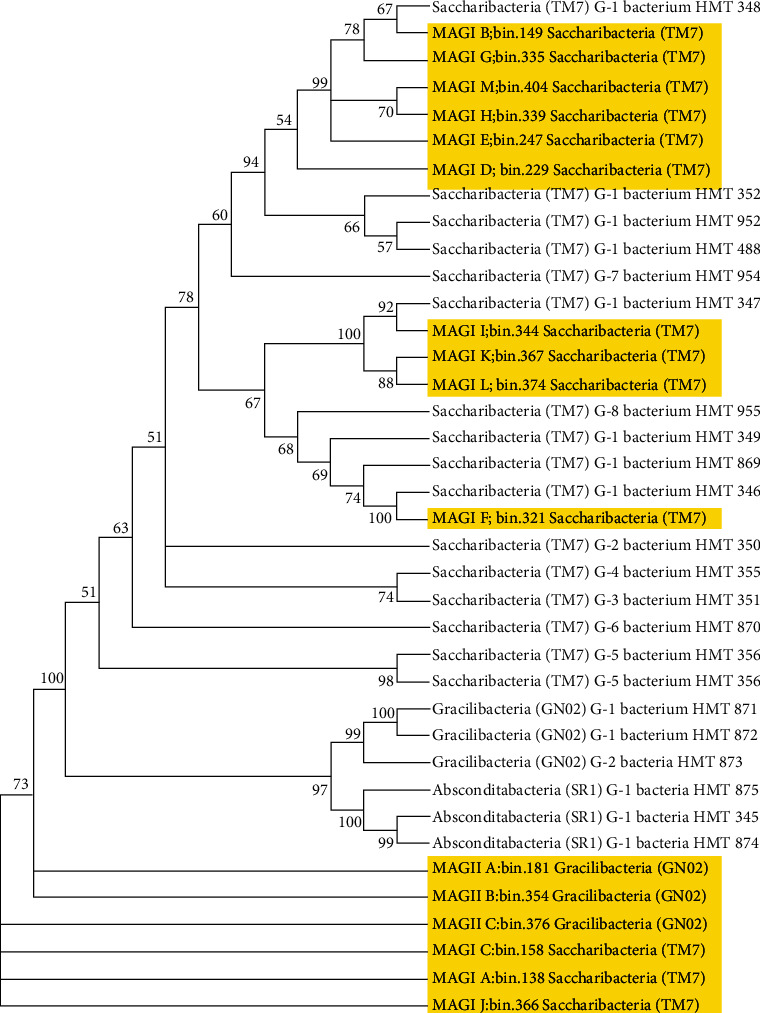
Phylogenetic relationships within the CPR of assembled in the study and derived from public ribosomal databases and available genomes using maximum-likelihood 16S rRNA. Highlights show CPR assembled for this study.

We used 16S SSU rRNA gene reads extracted from the sixteen CPR MAGs and twenty-two representative CPR genomes to generate a 16S rRNA phylogenetic tree by using the maximum-likelihood method to further verify the classification of MAGs (see Figure 1). Analysis of the phylogenetic tree showed MAG I.A I.C I.J and MAG II.C have a distant lineage with all other species. It may mean they correspond to entirely “novel” genomes that we found. There are also some species with particularly similar lineage, such as MAG I.B and TM7 G-1 HMT348, MAG I.I and TM7 G-1 HMT347, and MAG I.F and TM7 G-1 HMT346 suggesting that they may be the same flora. Most MAGs have a pedigree relationship with the reference genomes at a medium distance.

The abovementioned results indicate that all 16 new MAGs are derived from the CPR organisms, including thirteen TM7 and three GN02. There are many research studies on TM7 but still few studies on GN02. TM7 is a kind of highly diversified bacteria in the world, which was first identified in peat bog in Germany [43]. TM7 can be found in a variety of globally distributed environments, including fresh water, seawater, hot spring, and soils [44]. It has also been detected in many parts of human bodies, including skin [45], the distal esophagus [46] and intestinal tract [4], which is especially common in the oral cavity [47]. It is considered that TM7 is a common and possibly permanent component of the oral flora, which has the ability to maintain growth under both healthy and severe disease state conditions [48].

### 3.3. The Quality of MAGs Was Higher Than the Results in the Original Literature

Quast was used to determine the quality of the sixteen MAGs and five original literature's MAGs including three TM7 MAGs and two GN02 MAGs for further comparison. The contig N50 in this study was 28,065 significantly larger than the N50 of 5,586 in the original literature. All our MAGs contain a total of 215 contigs greater than 50,000 bp in length. However, the number of contigs larger than 50,000 bp in the original literature is zero (see Table 2). Compared with the original literature, we have obtained more MAGs and higher quality. Then, we compared MAG pairwise using MAUVE and JSpecies to calculate ANI. At least 95% (ANI) is considered the identification standard of the same species. Species identical to those in the original literature were not found but there are some similar species (see Tables 3 and 4). MAGs that are not matched to the original literature are newly excavated CPR genomic information.

### 3.4. The Difference of CPR in Caries and Caries-Free Groups

The results of this study showed that in the caries-free group, the TM7 and the GN02 were second in abundance to the major phyla, indicating that CPR is an important component of the oral flora. The results of this study also showed that the CPR at the species level were mainly *Candidatus Saccharibacteria* oral taxon TM7 of phylum TM7 and *Candidatus Gracilibacteria* bacterium of phylum GN02, both of which had higher abundance at the oral flora species level, which further proved the importance of CPR in the oral microbiota. TM7 and GN02 were common to both caries and caries-free groups and were detected in all samples, indicating that CPR are members of the oral “core microbiome,” and they may play an important role in the stability and function of the oral microecological environment. Based on the annotation level of the existing database, LefSe difference analysis showed that there was no significant difference between the bacteria of phylum TM7 and phylum GN02 in the caries and healthy groups in general. In this study, we followed up with an in-depth analysis of CPR obtained by genome assembly and binning, and the results showed that there were differences in CPR at the level of unknown strains in the caries and caries-free groups.

**Figure 2:**
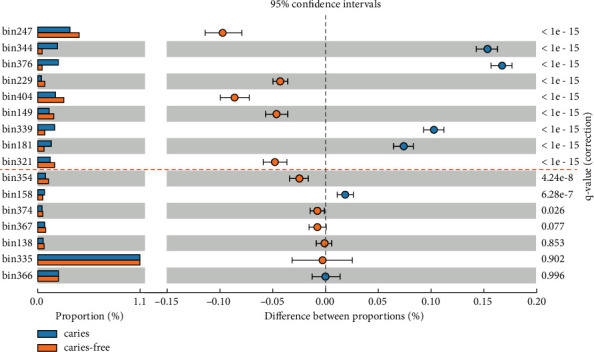
Comparison of relative abundance of each CPR MAGs between caries and caries-free groups. CPR MAGs above the dotted line are considered to be significantly different.

**Figure 3:**
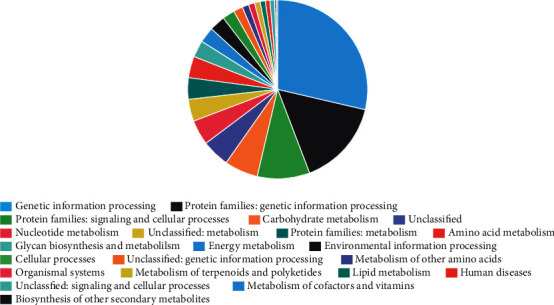
The functional predictions of proteins encoded in the genomes of MAG I.I.

**Figure 4:**
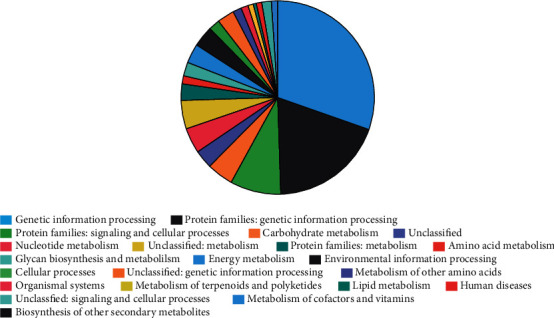
The functional predictions of proteins encoded in the genomes of MAG II.C.

We used STAMP to calculate the relative abundance for each CPR MAG in the caries group and caries-free group (see Figure 2). The relative abundance for MAG I.I and MAG II.C are significantly higher in caries than in caries-free groups. We selected the two MAGs with the greatest difference to study the functional predictions of the encoded proteins in their genomes (see Figures 3 and 4). Most of the functional genes within the two genomes encoded proteins are related to genetic information processing, in addition to signaling and cellular processes. Genetic information processing and cellular processes are of great significance to all microorganisms. The two of them together comprised 38.1% and 38.8% of the genes in the MAG I.I and MAG II.C, respectively, suggesting the importance of cell motility, cell envelope biogenesis, and signal transduction for these organisms. These results are consistent with the phenotypic characteristics of TM7 isolates. A recent study showed that TM7 species in the human oral cavity had undergone morphological changes, which showed that they changed from ultramicrococcus to slender cells in response to environmental cues including oxygen levels and nutritional status [49]. These cues could also be involved in cell signal transduction and activation of the cell movement pathway.

Compared with other bacterial genomes [50], genes involved in metabolism only account for a small proportion in the newly constructed CPR genomes, with 25.2% and 27.2% in the MAG I.I and MAG II.C. These results are consistent with the hypothesis that the biological metabolic capacity of CPR lineage is limited. Some previous studies have shown that the genomes of CPR organisms lack genes for the biosynthesis of most nucleotides, amino acids, lipids, and vitamins. In addition, the genomes lack most of the genes for the tricarboxylic acid cycle and electron transport chain components. In view of the fact that the metabolic capacities of some CPR organisms have recently been extended to members of the Parcubacteria, it is based on new genomes encoding putative components of the dissimilatory nitrate reduction to ammonia pathway [51, 52], and those genomes involved in hydroxylamine oxidation [51]. Metaproteomic analyses also showed that fermented CPR may play an important role in the hydrogen and carbon cycle of underground ecosystems [15, 53]. In this study, we identified genes encoding various transporters and enzymes in these two newly constructed genomes with significant differences, including genes encoding glycolysis-specific proteins of triosephosphate isomerase, glyceraldehyde-3-phosphate dehydrogenase, 6-phosphofructokinase, and enolase. We did not find a complete tricarboxylic acid cycle pathway in either of these MAGs. These results are consistent with previous hypotheses, that is, cardiopulmonary resuscitation organisms may show a symbiotic lifestyle and only have a part of the metabolic pathway. However, because the genome is incomplete, they may lack a complete metabolic pathway.

## 4. Conclusions

In conclusion, we identified and characterized CPR organisms that were difficult to culture using metagenomic sequencing approaches via a metagenomic approach. Using this approach, we reconstructed more and higher quality CPR MAGs from human oral supragingival plaque with or without dental caries using metagenomic data and confirmed the affiliation of genomes. By comparing the abundance and function differences of each MAG between caries and healthy subjects, two MAGs are suspected to be related to caries activity. In order to better understand the characteristics of the CPR organisms, more efforts should be conducted to develop a method to cultivate them in the future.

## Figures and Tables

**Table 1:** Basic information about CPR bins.

Identifier	Family	Genus	Species	Completeness	Contamination	No. of contigs
bin.138 (MAGI.A)	Unclassified Saccharibacteria	*Candidatus saccharimonas*	*Candidatus saccharibacteria* oral taxon TM7	91.67	132.01	135
bin.366 (MAGI.J)				91.67	50.00	28
bin.158 (MAGI.C)				91.67	88.02	44
bin.339 (MAGI.H)				91.67	66.67	58
bin.404 (MAGI.M)				91.67	66.67	32
bin.335 (MAGI.G)				91.67	72.19	19
bin.344 (MAGI.I)				91.67	0.85	28
bin.149 (MAGI.B)				89.47	61.46	162
bin.374 (MAGI.L)				81.03	76.88	75
bin.321 (MAGI.F)				66.67	30.95	104
bin.229 (MAGI.D)				65.95	59.65	144
bin.247 (MAGI.E)				65.53	43.90	13
bin.367 (MAGI.K)				62.92	22.81	29

bin.376 (MAGII.C)	Unclassified Gracilibacteria	Unclassified *Gracilibacteria*	*Candidatus gracilibacteria* bacterium	94.64	7.27	15
bin.181 (MAGII.A)				93.10	80.22	963
bin.354 (MAGII.B)				62.07	6.90	34

**Table 2:** Comparison of CPR bins' features in the original literature and in this study.

	CPR bins in the original literature	CPR bins in this study
No. of bins	5	16
No. of contigs (≥50000 bp)	0	215
N50	5586	28065
N75	3037	7917

**Table 3:** ANI values for assembled CPR bins and original CPR bins (TM7).

Identifier	RBJO01	RBJP01	RBJQ01
bin.138	73.56	67.09	67.10
bin.366	70.32	67.07	66.33
bin.158	76.34	67.12	68.63
bin.229	69.27	91.12	66.05
bin.339	70.10	66.77	94.28
bin.344	69.18	64.87	94.53
bin.404	81.95	67.02	69.55
bin.149	81.41	67.09	69.27
bin.321	82.86	66.02	69.61
bin.247	79.23	66.84	68.85
bin.367	72.85	65.77	87.21
bin.335	71.05	67.00	66.41
bin.374	68.31	66.29	94.06

**Table 4:** ANI values for assembled CPR bins and original CPR bins (GN02).

Identifier	RBJV01	RBJW01
bin.181	92.03	63.46
bin.376	94.65	64.71
bin.354	67.10	85.06

## Data Availability

The data used to support the findings of the study are available from the corresponding author upon request. The raw data are available through accession numbers SRR6865436 to SRR6865523, and the genome bines are available through biosample accession numbers SAMN17152884 to SAMN17152899.

## Supplementary Materials

Supplementary Materials Human subject metadata: metadata for human subjects used in this analysis.
